# Prevalence and Predictors of Thyroid Dysfunction in Patients with HIV Infection and Acquired Immunodeficiency Syndrome: An Indian Perspective

**DOI:** 10.1155/2015/517173

**Published:** 2015-12-22

**Authors:** Neera Sharma, Lokesh Kumar Sharma, Deep Dutta, Adesh Kisanji Gadpayle, Atul Anand, Kumar Gaurav, Sabyasachi Mukherjee, Rahul Bansal

**Affiliations:** ^1^Department of Biochemistry, Post Graduate Institute of Medical Education & Research (PGIMER) and Dr. Ram Manohar Lohia (RML) Hospital, 1 Baba Kharak Singh Marg, New Delhi 110001, India; ^2^Department of Endocrinology, Post Graduate Institute of Medical Education & Research (PGIMER) and Dr. Ram Manohar Lohia (RML) Hospital, 1 Baba Kharak Singh Marg, New Delhi 110001, India; ^3^Post Graduate Institute of Medical Education & Research (PGIMER) and Dr. Ram Manohar Lohia (RML) Hospital, 1 Baba Kharak Singh Marg, New Delhi 110001, India; ^4^Anti-Retroviral Therapy Clinic, Post Graduate Institute of Medical Education & Research (PGIMER) and Dr. Ram Manohar Lohia (RML) Hospital, 1 Baba Kharak Singh Marg, New Delhi 110001, India

## Abstract

*Background*. Predictors of thyroid dysfunction in HIV are not well determined. This study aimed to determine the prevalence and predictors of thyroid dysfunction in HIV infected Indians.* Methods*. Consecutive HIV patients, 18–70 years of age, without any severe comorbid state, having at least 1-year follow-up at the antiretroviral therapy clinic, underwent clinical assessment and hormone assays.* Results*. From initially screened 527 patients, 359 patients (61.44 ± 39.42 months' disease duration), having good immune function [CD4 count >200 cell/mm^3^: 90.25%; highly active antiretroviral therapy (HAART): 88.58%], were analyzed. Subclinical hypothyroidism (ScH) was the commonest thyroid dysfunction (14.76%) followed by sick euthyroid syndrome (SES) (5.29%) and isolated low TSH (3.1%). Anti-TPO antibody (TPOAb) was positive in 3.90%. Baseline CD4 count had inverse correlation with TPOAb after adjusting for age and body mass index. Stepwise linear regression revealed baseline CD4 count, TPOAb, and tuberculosis to be best predictors of ScH after adjusting for age, weight, duration of HIV, and history of opportunistic fungal and viral infections.* Conclusion*. Burden of thyroid dysfunction in chronic HIV infection with stable immune function is lower compared to pre-HAART era. Thyroid dysfunction is primarily of nonautoimmune origin, predominantly ScH. Severe immunodeficiency at disease onset, TPOAb positivity, and tuberculosis were best predictors of ScH.

## 1. Introduction

Dramatic improvements in survival following institution of highly active antiretroviral therapy (HAART) have given rise to increased occurrence of endocrinopathies in HIV infected patients in the last two decades, which are associated with significant morbidity. Thyroid dysfunction is among the commonest endocrinopathies in HIV. Undiagnosed thyroid dysfunction, even subclinical hypothyroidism, is associated with significant morbidity and poor quality of life [1–4]. Subtle thyroid dysfunction is common, believed to occur in as many as 35% of all HIV infected individuals [5–7]. In contrast overt thyroid dysfunction is less common, believed to involve 1-2% of all patients [6, 7]. Prevalence of overt primary hypothyroidism in the general population and HIV infected individuals from different studies across the globe has been reported to be 0.3% and 0–2.6%, respectively [6–9]. Similarly the prevalence of subclinical hypothyroidism has also reported to be higher in HIV infected individuals as compared to the general population (4.3% versus 3.5–12.2%) in different studies [6–9]. Most of the hypothyroidism in general population is believed to be of autoimmune etiology, in contrast to HIV, where a majority is believed to be of nonautoimmune origin [8, 9]. Stavudine has been linked with hypothyroidism in some studies [8, 9]. However the data on the prevalence of thyroid dysfunction in HIV infected patients from India is scant. Also factors, which determine the occurrence of thyroid dysfunction, have not been well determined. Patients with HIV infection often have associated comorbidities like infections, malignancies that are associated with significant stress, which may have an impact on hypothalamic-pituitary-thyroid axis making interpretation of thyroid function tests difficult. Hence the aim of this study was to determine the prevalence and predictors of the entire spectrum of thyroid dysfunction (overt hypothyroidism, subclinical hypothyroidism, subclinical hyperthyroidism, overt hyperthyroidism, and sick euthyroid syndrome) in stable ambulatory patients with HIV infection.

## 2. Methods

Antiretroviral therapy (ART) clinic at our institute has been functional since 2004, established by the National AIDS Control Organization (NACO), India, and the World Health Organization (WHO). The clinic provides for all the necessary investigations, medications (including highly active antiretroviral therapy [HAART]), counseling, and education to all patients with HIV infection. Consecutive ambulatory patients, 18–70 years of age, with serologically documented HIV infection, in stable clinical condition without any acute, severe illness, attending the ART clinic of our hospital were considered. Severely ill patients with multiple comorbid states, which would warrant hospital admission, were excluded. Patients with vitamin-D and/or calcium supplementation in the last 6 months were excluded. Patient records were reviewed and patients having clinical data of at least 1 year of follow-up were further evaluated. Patients whose CD4 cell count records at diagnosis and at least one follow-up (6–12 months after diagnosis) available were included in the study. The study protocol was explained to the considered patients and only those who gave informed written consent were included. The institutional ethics committee approved the study protocol. The period of the study was from August 2014 till September 2015.

Data were collected from the patients and their records regarding the duration of diagnosis of HIV infection and details of HAART. Data was also collected regarding past or current evidence of infections including opportunistic infections (bacterial, viral, and fungal). History of clinical features suggestive of hypothyroidism or hyperthyroidism was taken. All patients underwent detailed clinical assessment, including anthropometry. The patients were called the subsequent day in fasting state for blood sampling. Blood samples of 5 mL each were collected in plain and EDTA Vacutainer (Becton Dickinson). Serum was separated from blood collected in plain Vacutainer and processed immediately for routine biochemical analysis and one aliquot of serum was stored at −20 degrees' Celsius for specific immunological (hormonal) assays. EDTA sample was processed for hematological analysis.

Chemiluminescent microparticle immunoassay (VITROS ECiQ Immunodiagnostic System, Johnson & Johnson, USA) was used for estimation of free tri-iodothyronine (fT3), free tetraiodothyronine (fT4), thyroid stimulation hormone (TSH), and 25-hydroxy-vitamin-D. FT3 assay had analytical sensitivity of 0.50 pg/mL, analytical range of 0.50–22.8 pg/mL, and intra- and interassay coefficient of variation (CV) of 2.2% and 6.3%, respectively. FT4 assay had analytical sensitivity of 0.07 ng/dL and analytical range of 0.07–6.99 ng/dL with intra- and interassay coefficient of variation (CV) of 2.4% and 5.8%, respectively. TSH assay had analytical sensitivity of 0.015 mIU/L and analytical range of 0.015–100 mIU/L with intra- and interassay coefficient of variation (CV) of 3.3% and 7.2%, respectively. Serum 25-hydroxy-vitamin-D (25OHD) assay had analytical sensitivity of 8.0 ng/mL, analytical range of 8–150 ng/mL, and intra- and interassay coefficient of variation (CV) of 3.4% and 5.5%, respectively. Antithyroid peroxidase antibody (anti-TPO) levels were measured by solid phase immunosorbent assay (AutoSTAT II anti-TPO, HYCOR Biomedical) and had analytical sensitivity of 1.81 U/mL and analytical range of 35–1500 U/mL with intra- and interassay coefficient of variation (CV) of 3.7% and 7.5%, respectively. CD4 cell count was performed using flow cytometry (Becton Dickinson Immunocytochemistry Systems, San Jose, CA). Serum calcium, phosphate, alkaline phosphate, and renal function tests were done using clinical chemistry autoanalyzer based on dry chemistry microslide technology (VITROS 350 chemistry system, Johnson & Johnson, USA).

The reference range of fT3, fT4, and TSH in our laboratory is 2–4.4 pg/mL, 0.6–2.2 ng/dL, and 0.5–5 mIU/L, respectively. Anti-TPO antibody titer <50 IU/mL is considered to be negative or normal. In accordance with previous literature, primary overt hypothyroidism was defined as patients having elevated TSH along with low fT4 [5, 6]. Subclinical hypothyroidism was defined as patients having elevated TSH along with normal thyroid hormone levels. Subclinical hyperthyroidism was defined as patients having suppressed TSH levels with normal thyroid hormone levels [5, 6]. Overt hyperthyroidism was defined as patients having suppressed TSH levels along with elevated fT4 and/or fT3. Sick euthyroid syndrome, a physiological adaptive phenomenon to conserve energy during periods of extreme stress and infection, was defined as patients having isolated low fT3 or low fT4 with low fT3 levels, along with low or normal TSH levels [10]. Euthyroidism was defined as clinically asymptomatic patients having normal fT3, fT4, and TSH levels. It has been reported that 1.3–6.8% HIV infected patients have isolated low TSH with normal thyroid hormone levels [11]. Hence, patients with normal fT3 and fT4 with low TSH were grouped separately. A few studies have also noted isolated low fT4 (up to 6.8% patients), a pattern distinct from sick euthyroid syndrome in patients with HIV infection, which is believed to be due to an alternation in hypothalamic-pituitary-thyroid axis [6]. Hence these patients were also grouped separately in our study. Serum 25-hydroxy-vitamin-D (25OHD) levels ≥30 ng/mL were defined as vitamin-D sufficiency, 20–30 ng/mL as vitamin-D insufficiency, and <20 ng/mL as vitamin-D deficiency [12].

Immune reconstitution inflammatory syndrome (IRIS) in HIV infected patients is characterized by clinical deterioration in a patient secondary reestablishment of immunity following initiation of HAART [13]. It is usually observed in patients with low baseline CD4 count, which increases rapidly following initiation of HAART. HAART has been linked to increased occurrence of autoimmunity and autoimmune disorders [13, 14]. IRIS has been defined as an increased CD4 count above 200 cells/mm^3^ in patients who previously had CD4 counts lower than 100–200 cells/mm^3^ [15, 16]. Hence patients in our study with baseline CD4 counts less than 200 cells/mm^3^, which increased to >200 cells/mm^3^ at the first follow-up following initiation of HAART, were defined to have IRIS. Parameters like age, duration of HIV infection, occurrence of opportunistic infections, baseline and posttreatment CD4 counts, IRIS, antiretroviral drugs used, vitamin-D status, and autoimmunity (anti-TPO antibody) were evaluated for their role in predicting the occurrence of thyroid dysfunction.

Subtle thyroid dysfunction is common in HIV infected individuals, believed to involve as many as 35% of all individuals [5–7]. Hence for keeping a power of 80% and type-I error at 5%, it has been calculated that we need to recruit at least 246 patients in our study for accurate assessment of thyroid dysfunction.

## 3. Statistical Analysis

Normality of the distribution of variables was assessed using the Kolmogorov-Smirnov test. Independent *t*-test and Wilcoxon rank sum test were done for normally distributed and skewed variables, respectively. Chi-square tests were used for categorical variables. Pearson's or Spearman's correlation coefficient was calculated for normally distributed and skewed variables, respectively. Multiple linear regression analyses were done to determine variables that independently influenced the occurrence of thyroid dysfunction after adjusting for factors in different models. A *P* value <0.05 was considered statistically significant. SPSS version 20 was used for analyses.

## 4. Results

Five hundred and twenty-seven consecutive patients with HIV infection were screened at the ART clinic, of which 370 patients who fulfilled all criteria were considered for inclusion into the study. Reasons for exclusion from the study included severe associated illness (*n* = 23), multiple comorbid states like chronic liver disease, chronic kidney disease (*n* = 11), previous steroid use (*n* = 25), and history of calcium or vitamin-D supplementation in last 6 months (*n* = 41). Forty-six patients were excluded because of less than 1-year follow-up and 11 were excluded due to incomplete records. Of the 370 considered patients, 11 refused to consent to the study. Hence 359 patients (225 males and 134 females) who fulfilled all criteria and gave informed written consent underwent clinical assessment, hormonal evaluation, and analysis. Prevalence of vitamin-D deficiency (<20 ng/mL) and insufficiency (<30 ng/mL) among the study cohort was 55.71% (200/359) and 89.69% (322/359), respectively. Severe vitamin-D deficiency (<10 ng/mL) was observed in 9.19% (33/359) patients. At the time of diagnosis of HIV infection, 60.20% (216/359), 32.60% (117/359), and 7.20% (26/359) patients had CD4 count <200 cell/mm^3^, 200–500 cell/mm^3^, and >500 cell/mm^3^, respectively. The mean duration of HIV infection was 61.44 ± 39.42 months. Three hundred and nineteen (88.86%) patients were on HAART at the time of inclusion into the study. At the time of hormonal analysis, 9.75% (35/359), 58.50% (210/359), and 31.75% (114/359) patients had CD4 count <200 cell/mm^3^, 200–500 cell/mm^3^, and >500 cell/mm^3^, respectively. One hundred and forty-five patients (40.39%) had history of tuberculosis. None of the patients in this study had active tuberculosis at the time of recruitment. Six patients were on isoniazid and rifampicin at the time of recruitment as a part of maintenance phase of antitubercular therapy.

Subclinical hypothyroidism was the most common thyroid dysfunction observed in 53 (14.76%) patients. Sick euthyroid syndrome, isolated low TSH, and isolated low T4 were observed in 16 (4.45%), 11 (3.06%), and 3 (0.84%) patients, respectively. Overt hypothyroidism and hyperthyroidism were observed in 5 (1.39%) and 2 (0.01%) patients, respectively. Anti-TPO antibody titers were positive in 3.90% (14/359) patients (Table 1). Occurrence of thyroid dysfunction, especially sick euthyroid syndrome, was significantly more common in females than males (Table 1). Males were significantly older (*P* = 0.001) and had significantly lower BMI (*P* = 0.016), baseline CD4 count (*P* = 0.001), and current CD4 count (*P* = 0.001) along with significantly higher history of IRIS (*P* = 0.008) (Table 1).

Patients with history of IRIS were older (*P* = 0.049), were more likely to be males (*P* = 0.007), had lower BMI (*P* = 0.002), higher history of tuberculosis (*P* = 0.002), and higher use of protease inhibitors (*P* < 0.001), and had significantly lower baseline (*P* < 0.001) and current CD4 cell counts (*P* = 0.005) (Table 2). Serum fT3 was significantly higher in patients with history of IRIS (*P* = 0.036) (Table 2). The occurrence of different types of thyroid dysfunction was comparable in patients with history of IRIS as compared to those without (Table 2).

An inverse correlation was observed between baseline CD4 count (*P* = 0.031) and anti-TPO antibody titers, which persisted even after adjusting for age and body mass index (*P* = 0.032) (Table 3). Similarly an inverse correlation was observed in CD4 count at present with TSH levels, both at baseline (*P* = 0.043) and after adjusting for age and body mass index (*P* = 0.049) (Table 3). Stepwise linear regression analysis revealed that anti-TPO antibody titers and CD4 cell count at the time of initial diagnosis of HIV infection were the 2 best predictors of occurrence of subclinical hypothyroidism, at baseline (Model-1), after adjusting for age and duration of HIV infection (Model-2), and after adjusting for variables in Model-2 plus weight and history of opportunistic fungal and viral infections (Model-3) (Table 4). Increased anti-TPO antibody titers and lower baseline CD4 count were independent predictors of increased occurrence of subclinical hypothyroidism. Previous history of tuberculosis tended to be a good predictor of subclinical hypothyroidism later in life both at baseline (*P* = 0.084) and after adjusting for variables in Model-2 (*P* = 0.087) and Model-3 (*P* = 0.065) (Table 4).

## 5. Discussion

The occurrence of sick euthyroid syndrome among HIV infected patients is highly variable ranging from 1.3% to 11.6% in different studies [11, 16–19]. Stable, ambulatory, asymptomatic patients, with a large majority being on HAART (88.86%) with stable immune function (as evidenced by only 9.75% patients having CD4 count <200 cell/mm^3^ at the time of recruitment into this study and hormonal assessment) may explain the low occurrence of sick euthyroid syndrome in our study cohort. Heterogeneity in the disease profile of the patients evaluated in different studies (duration of infection, severity of immunodeficiency, in-patient versus out-patient, associated comorbidities, and functional status) may explain this variation.

A pilot study from central India reported a high prevalence of subclinical (30%) and overt hypothyroidism (10.66%) in a cohort of 150 HAART naïve newly diagnosed HIV infected patients [17]. Subclinical hypothyroidism was the commonest type of thyroid dysfunction observed in our study cohort documented in 14.76% patients. The prevalence is comparable to previous reports from other countries [6–9]. However this is in contrast to a previous report from our institute where a very high occurrence of subclinical hypothyroidism (53%) was documented in patients newly diagnosed with HIV infection [20]. It is important to highlight that the patients evaluated in that study were newly diagnosed with HIV infection, had advanced immunodeficiency (mean CD4 count: 147.1 ± 84 cell/mm^3^, 70.1% had CD4 count <200 cell/mm^3^), and were HAART naïve [20]. This is consistent with data reported from pre-HAART era and from patients newly diagnosed with HIV infection not on HAART, where the occurrence of thyroid dysfunction has been reported to be higher ranging from 10 to 40% [17, 21]. This is in contrast to our patients having mean disease duration of 5 years, with a large majority being on HAART (88.6%), being clinically stable, asymptomatic, and ambulatory with good immune function (90.25% having CD4 count >200 cell/mm^3^; 31.75% having CD4 count >500 cell/mm^3^).

Anti-TPO antibody titers, baseline CD4 counts, and history of tuberculosis were the best predictors of subclinical hypothyroidism in HIV infected patients in our study. Anti-TPO antibody positivity has previously been documented to be predictors of subclinical and overt hypothyroidism in normal and postpartum individuals [22, 23]. However it needs to be emphasized that, in spite of being a good predictor of subclinical hypothyroidism in our study, the prevalence of elevated anti-TPO antibody titers in our cohort was only 3.9%, which is lower than that observed in the general population (3–15%) [24]. A previous study of 642 normal individuals from northern India revealed the prevalence of anti-TPO positivity to be 21% [25].

The inverse relation observed between current CD4 count and TSH even after adjusting for variables highlights the possible link between increased immunodeficiency and elevated TSH. Further, in the setting of low prevalence of elevated anti-TPO antibody titers, low baseline CD4 count being a strong independent predictor of subclinical hypothyroidism highlights the importance of early immunodeficiency and HIV infection per se having an important role in the genesis of subclinical hypothyroidism later in life. It is likely that patients with lower CD4 counts had higher viral load. However, HIV viral load was not evaluated in our study and is a limitation of this report. Previous history of tuberculosis being an important independent predictor of hypothyroidism in our study also highlights the importance of immunodeficiency in the pathogenesis of thyroid dysfunction (as tuberculosis is more common in the immunodeficient state). A high rate of hypothyroidism (54%) was reported from 69 HIV infected patients with multidrug resistant (MDR) tuberculosis from Mumbai, India [26]. Use of rifampicin, para-aminosalicylate (PAS), and ethionamide for treating tuberculosis has been linked to the increased occurrence of hypothyroidism in these patients [26].

The inverse relationship observed between baseline CD4 count and anti-TPO antibody titers raises the possibility of increased immunodeficiency early in the course of disease being linked to increased occurrence of autoimmunity later. Increased autoantibody expression has previously been documented in patients of HIV infection with lower CD4+ cell counts, believed to be the result of a direct effect of the virus on endothelium, hematopoietic cells, and different tissues leading to enhancement of the cytotoxic activity of immune cells and autoantigen expression [27, 28]. Induction of immune restoration by HAART has been linked to thyroid dysfunction in some studies [29, 30]. This was primarily observed with regard to occurrence of hyperthyroidism and Graves' disease [17, 29, 30]. Graves' disease has most commonly been reported 12–36 months after HAART initiation [17]. Subclinical hyperthyroidism was not observed in our study. Also the occurrence of overt hyperthyroidism was too low in our study (0.01%) to be able to evaluate the factors responsible for their occurrence. In our study, IRIS was not a predictor of thyroid dysfunction.

In our study, none of the antiretroviral agents were predictors of occurrence of subclinical hypothyroidism. Studies evaluating the link between HAART and thyroid dysfunction have given conflicting results, with some but not all studies observing a link [6, 7]. A large cross-sectional study in 350 patients did not observe any link between HAART and occurrence of subclinical hypothyroidism [6]. Afhami et al. documented similar observations in a cross-sectional study of 85 HIV infected patients [31]. A higher than expected incidence of hypothyroidism (10.7 per 1000 treated patient years) but not hyperthyroidism (3.7 per 1000 treated patient years) was observed in a large study of 2437 HIV infected patients evaluated over 11-year period from 1995 to 2006 in United Kingdom [32]. Isolated low TSH was observed in 3.1% of patients in our cohort, which is in accordance with previous reports, which have documented a 1.3–6.8% prevalence of this condition [11]. The cause for this isolated low TSH is not well known. It may represent a spectrum of sick euthyroid syndrome. Increased endogenous cortisol tone, secondary to stress of HIV and associated comorbidities, may explain the isolated suppressed TSH levels [16]. In contrast to few previous reports, isolated low fT4 was uncommon in our study observed in only 0.84% of study patients. The cause for this isolated low fT4 is not well known. It is believed that these patients have intact response to TRH stimulation test in contrast to sick euthyroid syndrome [6, 33].

HIV infection has been linked to increased circulating levels of globulins including thyroid binding globulin, cortisol binding globulin, and sex-hormone binding globulin [34]. The cause for this phenomenon is not known. Hence total hormone (T3 and T4) may be falsely elevated in HIV patients. This was the rationale for measuring free T3 and free T4 in our study.

## 6. Conclusion

To summarize, it may be said that the burden of thyroid dysfunction in patients with chronic HIV infection with stable immune function (secondary to use of HAART) is lower compared to the pre-HAART era and newly diagnosed HAART naïve HIV infected patients. Subclinical hypothyroidism is the commonest type of thyroid dysfunction followed by sick euthyroid syndrome. Patients with more severe immunodeficiency (lower CD4 count) at disease onset and history of tuberculosis were more likely to have subclinical hypothyroidism later in life. Our study reiterated that thyroid dysfunction in HIV is primarily of nonautoimmune origin. However presence of elevated anti-TPO antibody titers in HIV was also independently associated with subclinical hypothyroidism similar to the normal population.

## Figures and Tables

**Table 1:** Clinical, biochemical, and thyroid function profile of males as compared to females with HIV infection.

Parameter	Males (*n* = 225)	Females (*n* = 134)	*P* value
Age (years)^a^	39 [49]	35 [39]	0.001
Duration of HIV infection (months)^a^	58 [168]	53 [137]	0.333
Body mass index (kg/m^2^)^a^	21.64 (4.88)	22.91 (4.09)	0.016
History of tuberculosis	97 (43.11%)	48 (35.82%)	0.173^#^
History of opportunistic fungal infections	4 (1.78%)	0	—
History of viral infections^*∗*^	7 (3.11%)	1 (0.75%)	0.150^#^
HAART	199	120	0.747^#^
Nature of HAART			
NRTI	198	120	0.654^#^
NNRTI	193	112	0.574^#^
PI	11	8	0.658^#^
IRIS	99	40	0.008^#^
CD4 cell count (at diagnosis)^a^	168 [1242]	195 [1922]	0.001
CD4 cell count (6–12 months after diagnosis)^a^	274 [947]	311 [1598]	0.010
CD4 cell count (at present)^a^	389 [1000]	458 [1355]	0.001
Free T3 (pg/mL)	3.51 (0.58)	3.49 (0.72)	0.741
Free T4 (ng/dl)	0.87 (0.16)	0.87 (0.28)	0.827
TSH (mIU/L)^a^	2.77 [18.57]	3.14 [26.9]	0.353
Anti-TPO antibody (U/mL)^a^	31 [175.75]	33 [164.7]	0.375
Anti-TPO positivity (>50 IU/L)	9 (4%)	5 (3.73%)	0.999^#^
Calcium (mg/dL)	9.17 (0.56)	9.19 (0.59)	0.812
Phosphate (mg/dL)	3.58 (0.59)	3.78 (0.60)	0.017
ALP (U/L)	133.2 (58.61)	118.16 (40.34)	0.045
25OHD (ng/mL)^a^	19.22 [75.70]	18.99 [61.5]	0.097
Euthyroidism	180 (80%)	89 (66.41%)	0.004
Overt primary hypothyroidism	2	3	0.291^#^
Subclinical hypothyroidism	31 (13.78%)	22 (16.41%)	0.495^#^
Subclinical hyperthyroidism	0	0	—
Overt hyperthyroidism	0	2 (1.49%)	—
Sick euthyroid syndrome/central hypothyroidism	6 (2.67%)	10 (7.46%)	0.033^#^
Low TSH with normal free T4	5 (2.22%)	6 (4.48%)	0.230^#^
Isolated low free T4	1 (0.44%)	2 (1.49%)	0.291^#^

All continuous variables expressed as mean (standard deviation); ^a^all nonnormally distributed variable expressed as median [range]; all discrete variables have been expressed as absolute numbers (percentage); Wilcoxon rank sum test was done for analysis; normally distributed continuous variables were analyzed using unpaired *t*-test; normality checked using Kolmogorov-Smirnov test; *P* < 0.05 considered statistically significant; ^#^ *P* value calculated using Chi-square test; ^*∗*^viral infections include hepatitis-B, hepatitis-C; HAART: highly active antiretroviral therapy; NRTI: nucleoside reverse transcriptase inhibitors; NNRTI: nonnucleoside reverse transcriptase inhibitor; PI: protease inhibitors; zidovudine (AZT), lamivudine (3TC), stavudine (d4T), and/or tenofovir (TDF) were the NRTIs received by the patients; nevirapine (NVP) or efavirinez (EFV) was NNRTIs received by the patients; atazanavir (ATV) or ritonavir (RTV) was the PI received by the patients; 25OHD: 25-hydroxy-vitamin-D; IRIS: immune reconstitution inflammatory syndrome; ALP: alkaline phosphate.

**Table 2:** Thyroid function profile of patients with immune reconstitution activation syndrome (IRIS) as compared to those without.

Parameter	History of IRIS (*n* = 139)	Patients without history of IRIS (*n* = 220)	*P* value
Age (years)^a^	39 [41]	36 [49]	0.049
Sex (male : female)	99 : 40	126 : 94	0.007^#^
Duration of HIV infection (months)^a^	56 [168]	55 [137]	0.667
Body mass index (kg/m^2^)^a^	21.58 [23.81]	21.94 [30.48]	0.002
History of tuberculosis	70 (50.36%)	75 (34.09%)	0.002^#^
History of opportunistic fungal infections	3 (2.16%)	1 (0.45%)	0.134^#^
History of viral infections^*∗*^	1 (0.72%)	7 (3.18%)	0.123^#^
HAART	124	195	0.867^#^
Nature of HAART			
NRTI	123	195	1^#^
NNRTI	118	187	1^#^
PI	16	3	<0.001^#^
CD4 cell count (at diagnosis)^a^	135 [192]	258 [1922]	<0.001
CD4 cell count (6–12 months after diagnosis)^a^	311 [657]	266 [1625]	0.144
CD4 cell count (at present)^a^	439 [1068]	389 [1355]	0.005
Free T3 (pg/mL)	3.53 (0.67)	3.41 (0.58)	0.036
Free T4 (ng/dL)	0.87 (0.23)	0.87 (0.17)	0.933
TSH (mIU/L)^a^	2.77 [26.72]	2.99 [19]	0.334
Anti-TPO antibody (U/mL)^a^	33 [175.75]	31 [144.59]	0.431
Anti-TPO positivity (>50 U/mL)	6 (4.32%)	8 (7.27%)	0.745
Calcium (mg/dL)	9.2 (0.56)	9.15 (0.59)	0.530
Phosphate (mg/dL)	3.71 (0.61)	3.53 (0.56)	0.031
ALP (U/L)	131 (60.41)	123.22 (39.26)	0.291
25OHD (ng/mL)^a^	18.23 [74.9]	19.7 [62]	0.335
Euthyroidism	107 (76.98%)	162 (73.64%)	0.477^#^
Overt primary hypothyroidism	1 (0.72%)	4 (1.82%)	0.386^#^
Subclinical hypothyroidism	18 (12.95%)	35 (15.91%)	0.441^#^
Subclinical hyperthyroidism	0	0	—
Overt hyperthyroidism	0	2 (0.91%)	—
Sick euthyroid syndrome/central hypothyroidism	7 (5.04%)	9 (4.09%)	0.666^#^
Low TSH with normal free T4	5 (3.60%)	6 (2.73%)	0.641^#^
Isolated low free T4	1 (0.72%)	2 (0.91%)	0.848^#^

All continuous variables expressed as mean (standard deviation); ^a^all nonnormally distributed variable expressed as median [range]; all discreet variables have been expressed as absolute numbers (percentage); Wilcoxon rank sum test was done for analysis; normally distributed continuous variables were analyzed using unpaired *t*-test; normality checked using Kolmogorov-Smirnov test; *P* < 0.05 considered statistically significant; ^#^ *P* value calculated using Chi-square test; ^*∗*^viral infections include hepatitis-B, hepatitis-C; HAART: highly active antiretroviral therapy; NRTI: nucleoside reverse transcriptase inhibitors; NNRTI: nonnucleoside reverse transcriptase inhibitor; PI: protease inhibitors; zidovudine (AZT), lamivudine (3TC), stavudine (d4T), and/or tenofovir (TDF) were the NRTIs received by the patients; nevirapine (NVP) or efavirinez (EFV) was NNRTIs received by the patients; atazanavir (ATV) or ritonavir (RTV) was the PI received by the patients; 25OHD: 25-hydroxy-vitamin-D; IRIS: immune reconstitution inflammatory syndrome; ALP: alkaline phosphate.

**Table 3:** Correlation between thyroid function parameters, CD4 cell count parameters, and vitamin-D in patients with HIV infection (*n* = 359).

	Free T3		Free T4		TSH		Anti-TPO antibody	
	Model-1	Model-2	Model-1	Model-2	Model-1	Model-2	Model-1	Model-2
CD4 count (baseline)^a^	0.016 (0.759)	−0.004 (0.947)	0.004 (0.939)	0.063 (0.271)	0.024 (0.646)	0.012 (0.836)	**−0.114 (0.031)**	**−0.123 (<0.032)**
CD4 count (6–12-month follow-up)^a^	−0.008 (0.878)	−0.37 (0.520)	0.041 (0.448)	0.105 (0.067)	−0.063 (0.237)	−0.094 (0.101)	−0.085 (0.113)	−0.086 (0.131)
CD4 count (current)^a^	−0.085 (0.123)	−0.098 (0.086)	0.012 (0.833)	0.045 (0.433)	**−0.111 (0.043)**	**−0.113 (0.049)**	0.001 (0.981)	−0.015 (0.789)
Serum 25OHD^a^	0.024 (0.651)	0.028 (0.625)	0.048 (0.368)	−0.01 (0.856)	−0.007 (0.900)	−0.003 (0.952)	−0.066 (0.216)	−0.098 (0.086)

Model-1: without adjustment for any variable; Model-2: after adjustment for age and body mass index; ^a^not normally distributed; Spearman's correlation coefficient calculated. All values expressed as correlation coefficient (*P* value); *P* < 0.05 considered statistically significant; TSH: thyroid stimulation hormone; TPO: thyroid peroxidase; T3: tri-iodothyronine; T4: tetraiodothyronine.

**Table 4:** Regression analysis showing parameters that are predictors of subclinical hypothyroidism in patients with HIV infection.

Variable	* *Model-1^a^		* *Model-2^b^		* *Model-3^c^	
	Standardized coefficient (*β*)	*P* value	Standardized coefficient (*β*)	*P* value	Standardized coefficient (*β*)	*P* value
Anti-TPO antibody	**0.245**	**<0.001**	**0.247**	**<0.001**	**0.239**	**<0.001**
CD4 count (baseline)	**−0.178**	**0.012**	**−0.175**	**0.018**	**−0.185**	**0.013**
CD4 cell count (6–12 months after diagnosis)^a^	−0.099	0.216	−0.094	0.242	−0.125	0.130
CD4 cell count (at present)^a^	−0.138	0.059	−0.138	0.062	−0.130	0.078
25OHD	−0.027	0.644	−0.025	0.679	−0.018	0.770
Tuberculosis	0.109	0.084	0.108	0.087	0.117	0.065
Lamivudine	−0.032	0.622	−0.030	0.653	−0.022	0.741
Stavudine	0.000	0.996	−0.010	0.903	−0.012	0.880
Tenofovir	−0.006	0.932	−0.001	0.991	0.009	0.904
Efavirinez	−0.126	0.053	−0.125	0.056	−0.125	0.056

Linear regression was initially performed with all parameters which are likely to influence serum testosterone levels [age, weight, duration of HIV infection, antithyroid peroxidase (TPO) antibody titer, baseline CD4 count, CD4 count (6–12 months after diagnosis), CD4 count at the time of recruitment into the study, hemoglobin, erythrocytic sedimentation rate, history of tuberculosis, opportunistic fungal infections, viral infections (hepatitis-B and hepatitis-C), serum 25-hydroxy-vitamin-D (25OHD), and individual antiretroviral agents received by the patient] to evaluate their role in the occurrence of subclinical hypothyroidism. ^a,b,c^Parameters with *P* < 0.2 were included into the stepwise forward line regression analysis without adjustment for any variables (Model-1), after adjustment for age and duration of HIV infection (Model-2) and after adjustment for variables in Model-2 plus weight and history of opportunistic fungal infections and viral infections (Model-3). Standardized coefficient (*β*): change in odds ratio with 1 unit change in predictor variable; for the tuberculosis variable, patients without tuberculosis were taken as reference group.
